# vol2Brain: A New Online Pipeline for Whole Brain MRI Analysis

**DOI:** 10.3389/fninf.2022.862805

**Published:** 2022-05-24

**Authors:** José V. Manjón, José E. Romero, Roberto Vivo-Hernando, Gregorio Rubio, Fernando Aparici, Mariam de la Iglesia-Vaya, Pierrick Coupé

**Affiliations:** ^1^Instituto de Aplicaciones de las Tecnologías de la Información y de las Comunicaciones Avanzadas (ITACA), Universitat Politècnica de València, Valencia, Spain; ^2^Instituto de Automática e Informática Industrial, Universitat Politècnica de València, Valencia, Spain; ^3^Departamento de Matemática Aplicada, Universitat Politècnica de València, Valencia, Spain; ^4^Área de Imagen Medica, Hospital Universitario y Politécnico La Fe, Valencia, Spain; ^5^Unidad Mixta de Imagen Biomédica FISABIO-CIPF, Fundación Para el Fomento de la Investigación Sanitario y Biomédica de la Comunidad Valenciana, Valencia, Spain; ^6^Centro de Investigación Biomédica en Red de Salud Mental, ISC III, València, Spain; ^7^Centre National de la Recherche Scientifique, Univ. Bordeaux, Bordeaux INP, Laboratoire Bordelais de Recherche en Informatique, UMR5800, PICTURA, Talence, France

**Keywords:** segmentation, brain, analysis, MRI, cloud

## Abstract

Automatic and reliable quantitative tools for MR brain image analysis are a very valuable resource for both clinical and research environments. In the past few years, this field has experienced many advances with successful techniques based on label fusion and more recently deep learning. However, few of them have been specifically designed to provide a dense anatomical labeling at the multiscale level and to deal with brain anatomical alterations such as white matter lesions (WML). In this work, we present a fully automatic pipeline (vol2Brain) for whole brain segmentation and analysis, which densely labels (*N* > 100) the brain while being robust to the presence of WML. This new pipeline is an evolution of our previous volBrain pipeline that extends significantly the number of regions that can be analyzed. Our proposed method is based on a fast and multiscale multi-atlas label fusion technology with systematic error correction able to provide accurate volumetric information in a few minutes. We have deployed our new pipeline within our platform volBrain (www.volbrain.upv.es), which has been already demonstrated to be an efficient and effective way to share our technology with the users worldwide.

## Introduction

Quantitative brain image analysis based on MRI has become more and more popular over the last decade due to its high potential to better understand subtle changes in the normal and pathological human brain. The exponential increase in the current neuroimaging data availability and the complexity of the methods to analyze them make the development of novel approaches necessary to address challenges related to the new “Big Data” paradigm (Van Horn and Toga, 2014). Thus, automatic, robust, and reliable methods for automatic brain analysis will have a major role in the near future, most of them being powered by cost-effective cloud-based platforms.

Specifically, MRI brain structure volume estimation is being increasingly used to better understand the normal brain evolution (Coupé et al., 2017) or the progression of many neurological pathologies such as multiple sclerosis (MS, Commowick et al., 2018) or Alzheimer's disease (Coupé et al., 2019).

The quantitative estimation of the different brain structure volumes requires automatic, robust, and reliable segmentation of such structures. As manual delineation of the full brain is unfeasible for routine brain analysis (this task is too tedious, time-consuming, and prone to reproducibility errors), many segmentation methods have been proposed over the years. Some of them were initially focused at the tissue level such as the famous Statistical Parametric mapping (SPM) (Ashburner and Friston, 2005). However, this level of detail may be insufficient to detect subtle changes in specific brain structures at early stages of the disease.

For example, hippocampus and lateral ventricle volumes can be used as early biomarkers of Alzheimer's disease. At this scale, also, cortical and subcortical gray matter (sGM) structures are of special interest for the neuroimaging community. Classic neuroimaging tools such as the well-known FSL package (Jenkinson et al., 2012) or Freesurfer (Fischl et al., 2002) have been widely used over the last 2 decades. More recently, multi-atlas label fusion segmentation techniques have been extensively applied, thanks to their ability to combine multiple atlas information minimizing mislabeling due to inaccurate registrations (Coupé et al., 2011; Wang and Yushkevich, 2013; Manjón et al., 2014; Romero et al., 2015).

However, segmentation of the whole brain into a large number of structures is still a very challenging problem even for modern multi-atlas based methods (Wang and Yushkevich, 2013; Cardoso et al., 2015; Ledig et al., 2015). The problems encountered are (1) the need of a large set of densely manually labeled brain scans and (2) the large amount of computational time needed to combine all those labeled scans to produce the final segmentation. Fortunately, a fast framework based on collaborative patch-matching was recently proposed (Giraud et al., 2016) to reduce the computational time required by multi-atlas patch-based methods.

More recently, deep leaning methods have also been proposed for brain structure segmentation. Those methods are mainly patch-based (Wachinger et al., 2018) or 2D (slice-based) (Roy et al., 2019) due to current GPU memory limitations. The current state-of-the-art whole brain deep learning methods are based on ensembles of local neural networks such as the SLANT method (Huo et al., 2019), or more recently the Assemblynet method (Coupé et al., 2020).

The aim of this study is to present a new software pipeline for whole brain analysis that we have called vol2Brain. It is based on an optimized multi-atlas label fusion scheme that has a reduced execution time, thanks to the use of our fast collaborative patch-matching approach, which has been specifically designed to deal with both normal appearing and lesioned brains (a feature that most of preceding methods ignored). This pipeline automatically provides volumetric brain information at different scales in a very simple manner through a web-based service not requiring any installation or technical requirements in a similar manner as previously done by our volBrain platform that since 2015 has processed more than 360,000 brains online worldwide. In the following sections, the new pipeline will be described, and some evidences of its quality will be presented.

## Materials and Methods

### Dataset Description

In our proposed method, we used an improved version of the full Neuromorphometrics dataset (http://www.neuromorphometrics.com), which consists of 114 manually segmented brain MR volumes corresponding to subjects with ages covering almost the full lifespan (from 5 to 96 years). Dense neuroanatomical manual labeling of MRI brain scans was performed at Neuromorphometrics, Inc., following the methods described in the study by Caviness et al. (1999).

The original MRI scans were obtained from the following sources: (1) the Open Access Series of Imaging Studies (OASIS) project website (http://www.oasis-brains.org/) (*N* = 30), (2) the Child and Adolescent NeuroDevelopment Initiative (CANDI) Neuroimaging Access Point (http://www.nitrc.org/projects/candi_share) (*N* = 13), (3) the Alzheimer's Disease Neuroimaging Initiative (ADNI) project website (http://adni.loni.usc.edu/data-samples/access-data/) (*N* = 30), (4) the McConnell Brain Imaging Center (http://www.bic.mni.mcgill.ca/ServicesAtlases/Colin27Highres/) (*N* = 1), and (5) the 20Repeats dataset (http://www.oasis-brains.org/) (*N* = 40).

Before manual labeling, all the images were preprocessed with an automated bias field inhomogeneity correction (Arnold et al., 2001) and geometrically normalized using three anatomical landmarks [anterior commissure (AC), posterior commissure (PC), and mid-sagittal point]. The scans were reoriented and resliced so that anatomical labeling could be done in coronal planes that follow the AC-PC axis. The manual outlining was performed using an in-house software called the NVM and the exact specification of each region of interest is defined in (1) Neuromorphometrics' General Segmentation Protocol (http://neuromorphometrics.com/Seg/) and (2) the BrainCOLOR Cortical Parcellation Protocol (http://Neuromorphometrics.com/ParcellationProtocol_2010-04-05.PDF). It has to be noted that the exact protocols used to label the scans evolved over time. Because of this, not all anatomical regions were labeled in every group (label number range: max = 142, min = 136).

### Dataset Correction

Right after downloading the Neuromorphometrics dataset, we performed a rigorous quality control of the dataset. We discovered that this dataset presented several issues that had to be corrected before using it.

#### Image Resolution, Orientation, and Size

After checking each individual file, we found that they had different acquisition orientations (coronal, sagittal, and axial). They also have different resolutions (1 ×1 ×1, 0.95 ×0.93 ×1.2, 1.26 ×1.24 ×12, etc.) and different volume sizes (256 ×256 ×307, 256 ×256 ×299, 256 ×256 ×160, etc.). To standardize them, we registered all image and corresponding label files to the MNI152 space using ANTS software, which resulted in a homogeneous dataset with axial orientation, 1 ×1 ×1 mm^3^ voxel resolution, and a volume size of 181 ×217 ×181 voxels. We also checked the image quality and we removed 14 cases from the original dataset that presented strong image artifacts and severe blurring effects. This resulted in a final dataset of 100 cases.

#### Inconsistent and Different Number of Labels

The selected 100 files from the previous step had 129 common labels from a total of 142 labels. After analyzing these 13 inconsistent labels, we decided to treat each of them in a specific manner according to the detected issue. Label file description assigns label numbers from 1 to 207. However, we found that labels 228, 229, 230, and 231 were present in some files. After checking them, we realized that labels 228 and 229 on the left corresponded to a right basal foreground (labels 75 and 76) and so we renumbered them. Labels 230 and 231 just represented few pixels in three of the cases and therefore were removed. Labels 63 and 64 (right and left vessel) were not present in all the cases (not always visible) and we decided to renumber them as a part of the putamen (labels 57 and 58), as they were located inside. We removed label 69 (optic chiasm) because it was not present in all the cases and its delineation was very inconsistent. Labels 71, 72, and 73 (cerebellar vermal lobules I-V, VI-VII, and VIII-X) were present in 74 of the 100 cases, and we decided to re-segment the inconsistent cases so that all the cases have these labels (details are given in the following section). Label 78 (corpus callosum) was only present in 25 cases, and we decided to relabel it as right and left white matter (WM, labels 44 and 45). Label 15 (5th ventricle) was very tiny and only present in a few cases (13); thus, it was relabeled as lateral ventricles (labels 51 and 52). Finally, we decided to add two new labels that we found important, i.e., external cerebrospinal fluid (CSF) (labeled as 1) and left and right WM lesions (labels 53 and 54). Details on how these labels were added are provided in the following section. After all the cleanup, the final dataset had a consistent number of 135 labels (refer to Appendix).

#### Labeling Errors

Once the dataset had a homogeneous number of labels, we inspected them to check their quality. After inspecting the dataset visually, we found that the boundaries of all the structures in sagittal and axial planes were very irregular. This is probably due to the fact that the original manual delineation was performed in the coronal plane. However, one of the main problems we found was the fact that cortical gray matter (cGM) was severely overestimated, and correspondingly, the CSF and WM were underestimated. This fact has been already highlighted by other researchers (Huo et al., 2017) who pointed out this problem in the context of cortical thickness estimation. The same problem arises in the cerebellum, although it is a bit less pronounced. To solve this problem (Huo et al., 2017), an automatic fusion of the original GM/WM maps was used, and partial volume maps were generated by the TOADS method (Bazin and Pham, 2008) to correct the cortical labels. In this study, we have followed a different approach based on the original manual segmentation and the intensity information.

**Figure 1:**
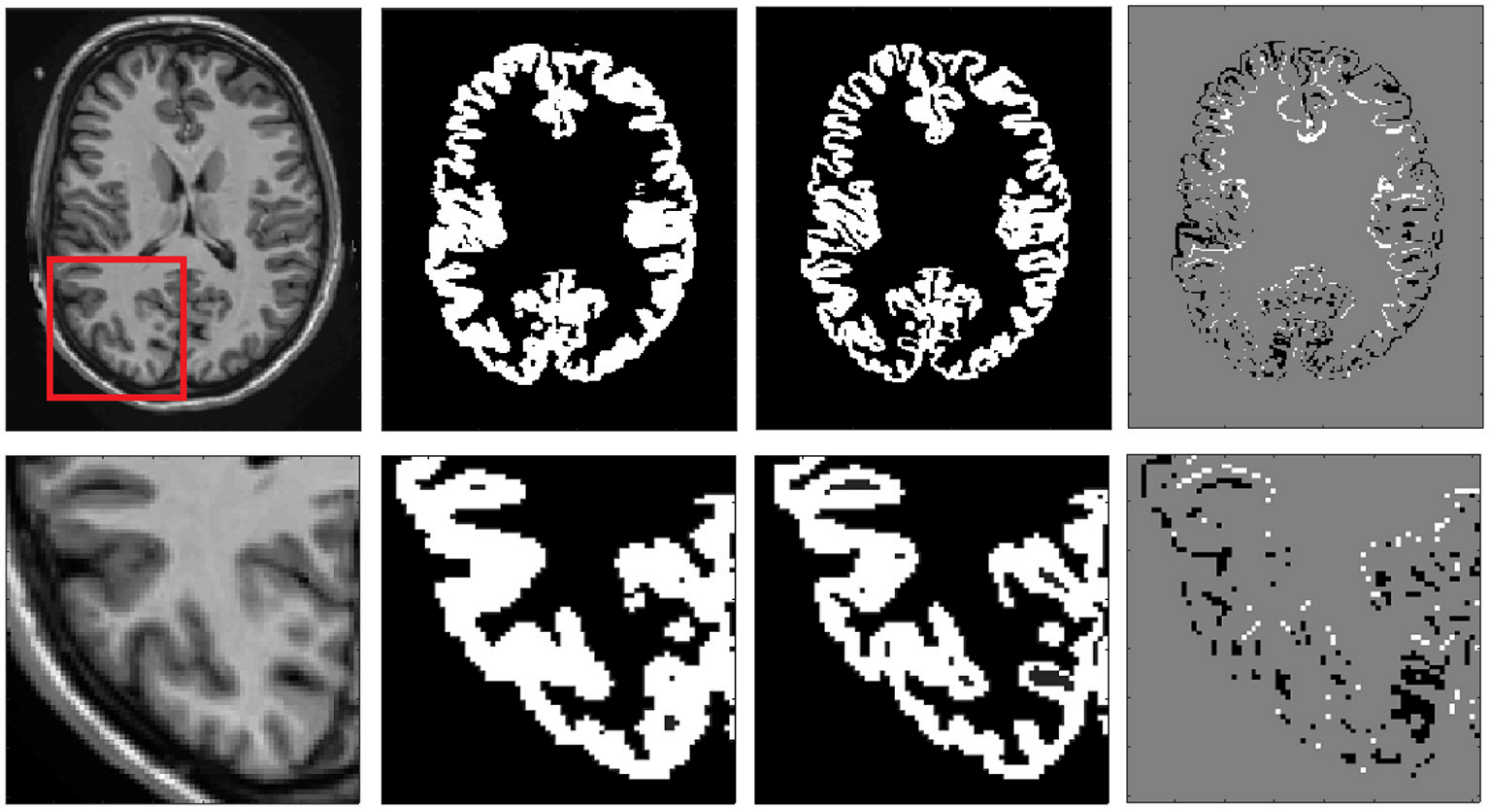
Example of cGM tissue correction. From right to left: Reference T1 image, original cGM map, corrected cGM map, and map of changes (white means inclusion and black means removal of pf voxels). In the bottom row, a close up is shown to better highlight the differences.

First, we combined all the 135 labels into seven different classes (CSF, cGM, cerebral white matter (cWM), sGM, cerebellar gray matter (ceGM), cerebellar white matter (ceWM), and brain stem (BS)]. External CSF was not labeled in the Neuromorphometrics dataset, so we added it using volBrain (Manjón and Coupé, 2016) (we copied CSF label to those pixels that had label 0 in the original label file). Then, the median value of cGM and cWM was estimated and used to generate the partial volume maps using a linear mixing model (Manjón et al., 2008). Voxels in the cGM and cWM interface were relabeled according to their partial volume content (e.g., a cGM voxel with a cWM partial volume coefficient bigger than its corresponding cGM partial volume coefficient was relabeled as cWM). The same process was repeated for the CSF/cGM interface, the ceGM/ceWM interface, and the ceGM/CSF interface. To ensure the regularity of the new label maps, each partial volume map was regularized using a non-local means filter (Coupé et al., 2018). Finally, each case was visually revised and small labeling errors were manually corrected using the ITK-SNAP software. Most of the corrections were related with cGM in the upper part of the brain, and misclassifications of WM lesions were termed as cGM and CSF-related corrections. Figure 1 shows an example of the cGM/cWM tissue maps before and after the correction.

**Figure 2:**
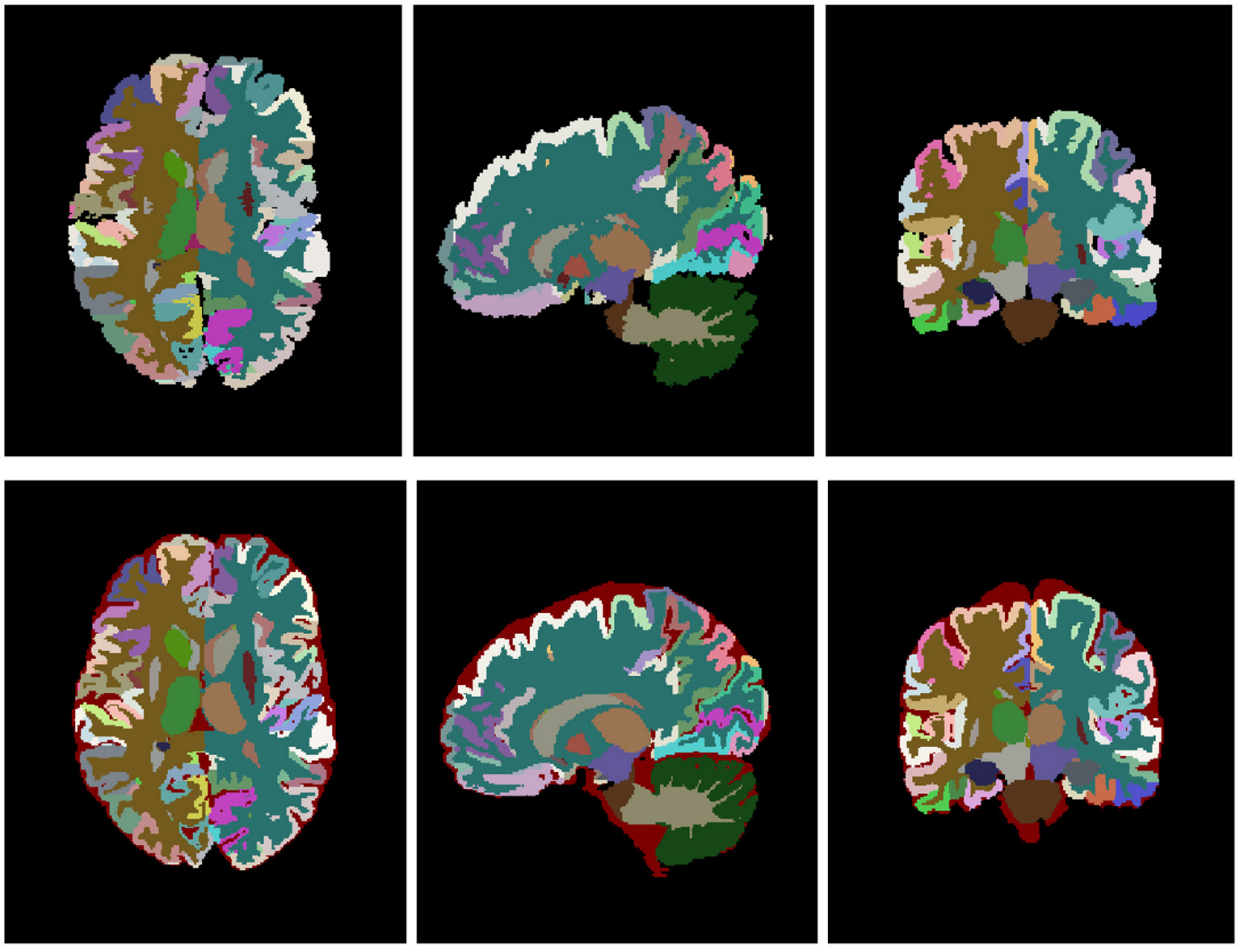
Top row shows the original labeling and bottom row shows the corrected labeling. Note that the external CSF label has been added to the labeling protocol.

After the tissue correction, the original structure labels were automatically relabeled to match the new tissue maps. Specifically, those voxels that kept the same tissue type before and after the correction kept their original labels and those that changed were automatically labeled according to the most likely label considering their position and intensity. Results were visually reviewed to assess its correctness and manually corrected when necessary. Finally, we realized that sGM structures showed important segmentation errors and we decided to re-segment them using volBrain automatic segmentation followed by manual correction when needed. Figure 2 shows an example of the final relabeling result.

#### LesionBrain Dataset

One of the main goals of the proposed pipeline was to make it robust to the presence of WM lesions that normally are misclassified as gray matter (GM) in pathological brains. To this end, we included not only healthy cases but also subjects with WM lesions in our library. Specifically, 32 of the 100 cases of the previously described Neuromorphometrics dataset had WM visible lesions with a lesion load ranging from moderate to severe. We are aware that WM lesions can appear anywhere in the brain, but it is also known that they have *a priori* probability to be located in the periventricular areas among others (Coupé et al., 2018).

We found though that the number of cases with lesions on the dataset was not enough to capture the diversity of WM lesion distribution, so we decided to expand the dataset using a manually labeled MS dataset. We previously used this dataset to develop a MS segmentation method (Coupé et al., 2018).

This dataset is composed of 43 patients with MS who underwent 3T 3D-T1w MPRAGE and 3D-Fluid-Attenuated Inversion Recovery (FLAIR) MRI. We used only the T1 images, as this is the input modality of our proposed pipeline. To further increase the size of the dataset, we included the left-right flipped version of the images and labels resulting in an extended dataset of 86 cases.

### Vol2Brain Pipeline Description

**Figure 3:**
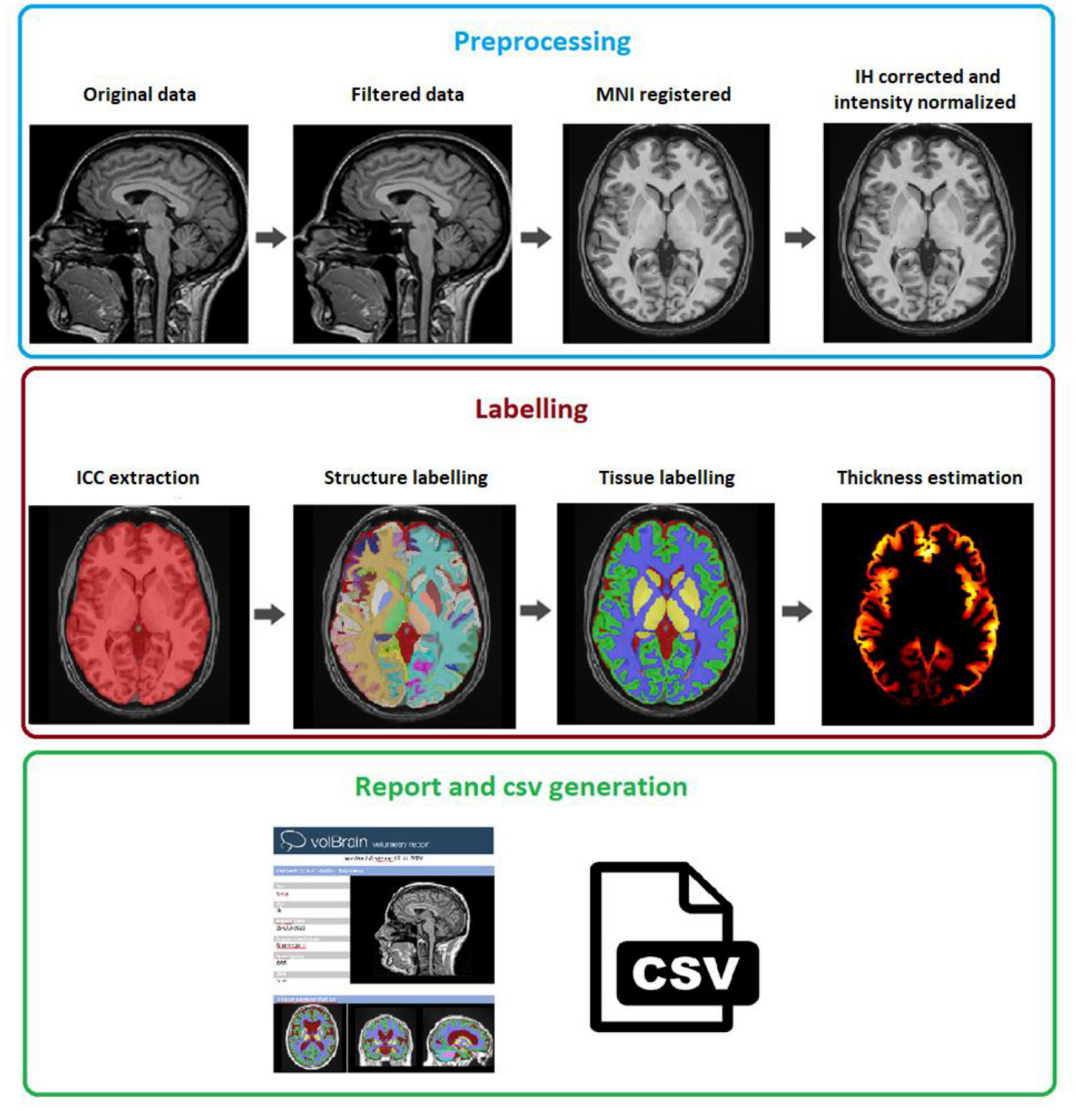
vol2Brain pipeline scheme. In the first row, the preprocessing for any new subject is presented. In the second row, the results of the ICC extraction, structure, and tissue segmentations jointly with the cortical thickness estimation are presented. Finally, in the third row, the volumetric information is extracted and presented.

The vol2Brain pipeline is a set of image processing tasks dedicated to improve the quality of the input data and to set them into a specific geometric and intensity space, to segment the different structures and to generate useful volumetric information (refer to Figure 3 for a general overview). The vol2Brain pipeline is based on the following steps:

1. Preprocessing
2. Multiscale labeling and cortical thickness estimation
3. Report and csv generation

#### Preprocessing

We have used the same preprocessing steps as those described in the volBrain pipeline (Manjón and Coupé, 2016), as it has been demonstrated to be very robust (based in our experience processing more than 360,000 subjects worldwide). This preprocessing consists of the following steps. To improve the image quality, first, the raw image is denoised using the Spatially Adaptive Non-Local Means (SANLM) filter (Manjón et al., 2010) and inhomogeneity is corrected using the N4 method (Tustison et al., 2010). The resulting image is then affinely registered to the Montreal Neurological Institute (MNI) space using the ANTS software (Avants et al., 2008). The image in the MNI space has a size of 181 ×217 ×181 voxels with 1 mm^3^ voxel resolution. Then, we used an inhomogeneity correction based on SPM8 (Ashburner and Friston, 2005) toolbox, as this model-based method has proven to be quite robust once the data are located at the MNI space. Finally, we normalized the images as per intensity by applying a piecewise linear tissue mapping based on the TMS method (Manjón et al., 2008) as described in the study by Manjón and Coupé (2016). It is worth to note that the library images were also normalized as per intensity using the described approach so that both library and the case to be segmented share a common geometrical and intensity space.

#### Multiscale Labeling and Cortical Thickness Estimation

After the preprocessing, the images are ready to be segmented and measured. This segmentation is performed in several stages.

##### ICC Extraction

The first step in the segmentation process is the intracranial cavity extraction (ICC). This is obtained using the NICE method (Manjón et al., 2014). NICE method is based on a multi-scale non-local label fusion scheme. Details of the NICE method can be found in the study by Manjón et al. (2014). To further improve the quality of the original NICE method, we have increased the size of the original volBrain template library from 100 to 300 cases using the 100 cases of the vol2Brain library and their left-right mirrored version.

##### Full Brain Structure Segmentation

The dense segmentation of the full brain is based on a multiscale version of the non-local patch-based label fusion technique (Coupé et al., 2011) wherein patches of the subject to be segmented are compared with patches of the training library to look for similar patterns within a predefined search volume to assign the proper label *v* as can be seen in the following equation:


(1) $$\begin{array}{l} v\left(x_{i}\right)\;=\;\frac{\overset{N}{\underset{s=1}{\sum }}\underset{j\in V_{i}}{\sum }w\left(x_{i},x_{s,j}\right)y_{s,j}}{\overset{N}{\underset{s\;=\;1}{\sum }}\underset{j\in V_{i}}{\sum }w\left(x_{i},x_{s,j}\right)} \end{array}$$

where *V*_*i*_ corresponds to the search volume, *N* is the number of subjects in the templates library, *y*_*s, j*_ is the label of the voxel *x*_*s, j*_ at the position *j* in the library subject *s*, and *w(x*_*i*_*, x*_*s, j*_*)* is the patch similarity defined as:


(2) $$\begin{array}{l} w\left(x_{i},x_{s,j}\right)\;=\;\exp^{\frac{-D_{i,j,s}}{h^{2}}} \end{array}$$

(3) $$\begin{array}{l} D_{i,j,s}\;=\;||P\left(x_{i}\right)\;-\;P\left(x_{s,j}\right)|{|}_{2}^{2} \end{array}$$

where P*(x*_*i*_*)* is the patch centered at *x*_*i*_, *P(x*_*s, j*_*)* is the patch centered at *x*_*j*_ in the templates, and ||.||_2_ is the normalized L2 norm (normalized by the number of elements) calculated from the distance between each pair of voxels from both patches *P(x*_*i*_*)* and *P(x*_*s, j*_*)*. *h* is a normalization parameter that is estimated from the minimum of all patch distances within the search volume.

However, exhaustive patch comparison process is very time-consuming (even in reduced neighborhoods, i.e., when the search volume V is small). To reduce the computational burden of this process, we have used a multiscale adaptation of the OPAL method (Giraud et al., 2016) previously proposed in the study by Romero et al. (2017), which takes benefit from the concept of Approximate Nearest Neighbor Fields (ANNF). To further speed up the process, we processed only those voxels that were segmented as ICC by the NICE method.

In patch-based segmentation, the patch size is a key parameter that is strongly related to the structure to be segmented and image resolution. It can be seen in the literature that multi-scale approaches improve segmentation results (Manjón et al., 2014). In the OPAL method (Giraud et al., 2016), independent and simultaneous multi-scale and multi-feature artificial neural networks (ANN) fields were computed. Thus, we have followed a multi-scale approach in which several different ANNs are computed for different patch sizes resulting in different label probability maps that have to be combined. In this study, two patch sizes are used, and an adaptive weighting scheme is proposed to fuse these maps (Equation 3).


(4) $$p(l)\;=\;\alpha \;p_{1\;}(l)\;+\;(1\;-\;\alpha )p_{2}(l)$$

where *p*_1_*(l)* is the probability map of patch-size 3 ×3 ×3 volxels for label *l, p*_2_*(l)* is the probability map of patch-size 5 ×5 ×5 voxels for label l, *p(l)* is the final probability map for label l, and α ϵ [0,1] is the probability mixing coefficient.

##### Systematic Error Correction

Any segmentation method is subject to both random and systematic errors. The first error type can be typically minimized by using bootstrapped estimations. Fortunately, the non-local label fusion technique estimates the voxel label averaging the votes of many patches, which naturally reduces the random classification error. Unfortunately, systematic errors cannot be reduced using this strategy, as they are not random. However, due to its nature, this systematic bias can be learned, and later, this knowledge can be used to correct the segmentation output (Wang and Yushkevich, 2013).

In the study by Romero et al. (2017), we proposed an error corrector method based on a patch-based ensemble of neural networks (PEC for Patch-based Ensemble Corrector) to increase the segmentation accuracy by reducing the systematic errors. Specifically, a shallow neural network ensemble is trained with image patches of sizes 3 ×3 ×3 voxels (fully sampled) and 7 ×7 ×7 voxels (subsampled by skipping two voxels at each dimension) from the T1w images, the automatic segmentations, a distance map value, and their x, y, and z coordinates at MNI152 space. The distance map we used is calculated for the whole structure as the distance in voxels to the structure contour. This results in a vector of 112 features that are mapped to a patch of manual segmentations of size 3 ×3 ×3 voxels. We used a multilayer perceptron with two hidden layers of size 83 and 55 neurons resulting in a network with a topology of 112 ×83 ×55 ×27 neurons. An ensemble of 10 neural networks was trained using a boosting strategy. Each new network was trained with a different subset of data, which was selected by giving a higher probability of selection to those samples that were misclassified in the previous ensemble. More details can be found in the original study (Romero et al., 2017).

#### Multiscale Label Generation

Once the full brain segmentation is performed, different scale versions were computed by combining several labels to generate more generic ones and allowing a multiscale brain analysis. The 135 labels were combined to create a tissue-type segmentation map, including eight different tissues [CSF, cGM, cWM, sGM, ceGM, ceWM, BS, and white matter lesions (WML)]. The cGM and cWM maps will be later used to compute the cortical thickness. Also, cerebrum lobe maps were created by combining cortical GM structures. These maps will be used later to compute the lobe-specific volumes and thickness.

#### Cortical Thickness Estimation

To estimate the cGM thickness, we have used the DiReCT method. DiReCT was introduced in the study by Das et al. (2009) and was made available in ANTs under the program named *KellyKapowski*. This method is based on the use of a dense non-linear registration to estimate the distance between the inner and the outer parts of the cGM. Cortical thickness per cortical label and per lobe were estimated from the thickness map and the corresponding segmentation maps (Tustison et al., 2014).

#### Report Generation

The output produced by the vol2Brain pipeline consists in a pdf and csv files. These files summarize the volumes and asymmetry ratios estimated from the images. If the user provides sex and age of the submitted subject, population-based normal volumes and asymmetry bounds for all structures are added for reference purposes. These normality bounds were automatically estimated from the IXI dataset (https://brain-development.org/ixi-dataset/), which contains almost 600 normal subjects covering most of the adult lifespan. We are aware that one of the most important sources of variability is the use of different scanners to build the normative values (although the use of our preprocessing reduces this variability). In the near future, we will extend the dataset to have a larger and more representative sample of the population as we already did for the volBrain pipeline (Coupé et al., 2017).

Furthermore, the user can access to its user area through volBrain website to download the resulting nifti files containing the segmentations at different scales (both in native and MNI space). An example of the volumetric report produced by vol2Brain is shown in Appendix.

## Experiments and Results

**Figure 4:**
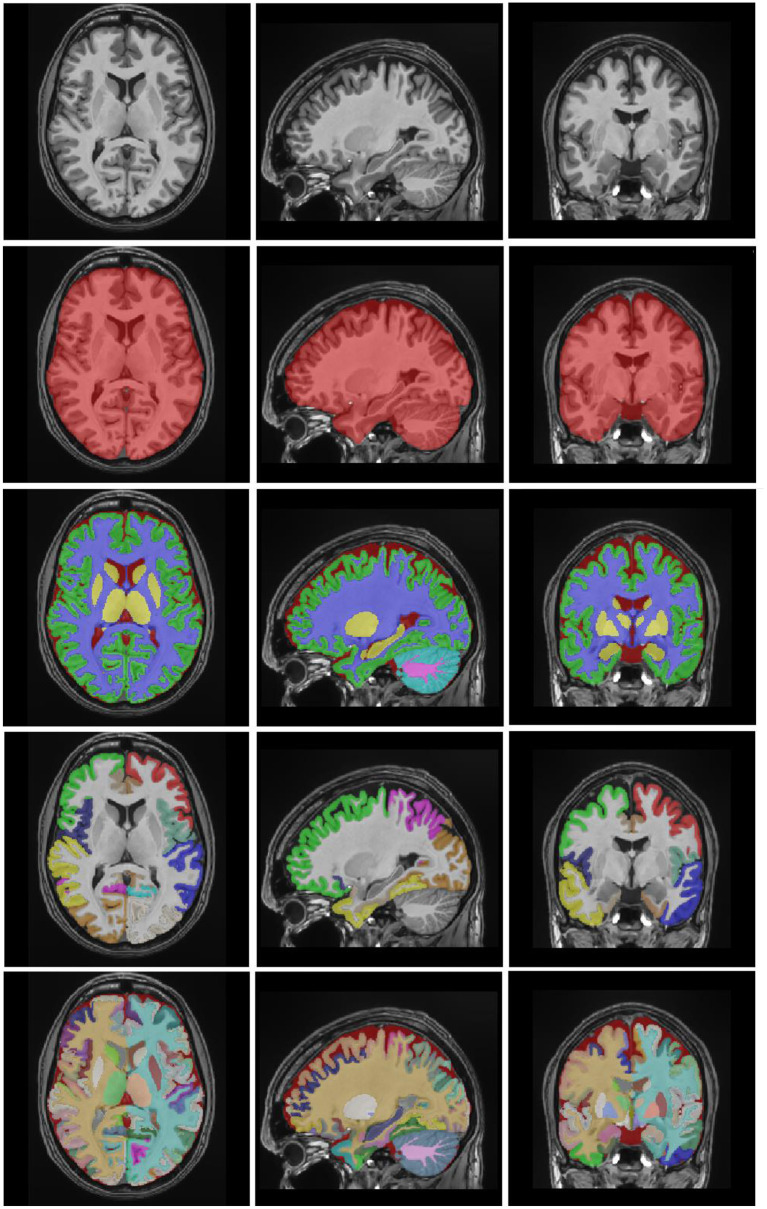
Example results of vol2Brain. T1 image, ICC mask, brain tissues, lobes, and structures.

In this section, some experimental results are shown to highlight the accuracy and reproducibility of the proposed pipeline. A leave-two-out procedure was performed for the 100 subjects of the library (i.e., excluding the case to be segmented and its mirrored version). In the dataset, there are 19 cases that were scanned and labeled twice for the purpose of reproducibility estimation. In this case, a leave-four-out procedure was applied to avoid any problem (i.e., excluding the case to be segmented and its mirrored version of the two acquisitions of the same subject). To measure the segmentation quality, the dice index (Zijdenbos et al., 1994) was computed by comparing the manual segmentations with the segmentations obtained with our method. A visual example of the automatic segmentation results is shown in Figure 4.

### Results

Since presenting dice results of the 135 labels would be impractical, we have decided to show the average results for cortical and non-cortical labels as done in previous studies (Wang and Yushkevich, 2013). In Table 1, the results of the proposed method are shown with and without the corrective learning step (PEC) to show the impact that this postprocessing has in the final results (it improved the results in all the cases).

**Table 1:** Proposed method dice results.

**Method**	**All labels**	**Cortical labels**	**Non-cortical labels**
Our method	0.8190 ± 0.0300	0.7912 ± 0.0397	0.8929 ± 0.0173
Our method + PEC	**0.8262** **±0.0257**	**0.7996** **±0.0347**	**0.8969** **±0.0157**

The mean dice is evaluated on all the considered labels (135 without background). *Best results highligthed in bold.

To further explore the results, we separated them by dataset, as it is well-known that results within the same dataset are normally better than across the datasets. This allows to explore the generalization capabilities of the proposed method. Results are summarized in Table 2. As can be seen, results of the OASIS dataset were the best among the datasets. This makes perfect sense, as precisely, this dataset is the largest. CANDI dataset showed the worst results. This dataset had the worst image quality, which somehow explains these results.

**Table 2:** Proposed method overall dice results for the full dataset and for each of the subsets.

**All** **(*N* = 100)**	**OASI**S **(*N* = 68)**	**CANDI** **(*N* = 6)**	**ADNI** **(*N* = 25)**	**COLIN** **(*N* = 1)**
0.8262 ± 0.0257	0.8353 ± 0.0233	0.7831± 0.0326	0.8111 ± 0.0142	0.8353

One of the objectives of the proposed method was to be able to deal with images with white mater lesions. This is fundamental, as if we do not take into account those regions, they are normally misclassified as a cGM or sGM (which also affects the cortical thickness estimation) (Dadar et al., 2021). The results of WM lesion segmentation are summarized in Table 3 (left and right lesions were considered together). We separated the results by lesion volume, as it is well-known that small lesions are more difficult to segment than the big ones (Manjón et al., 2018).

**Table 3:** Proposed method lesion dice results.

**Method**	**Small (*N* = 76)**	**Medium (*N* = 21)**	**Big (*N* = 3)**	**Avg (*N* = 100)**
Lesion	0.5767 ± 0.1486	0.8281 ± 0.0500	0.8467 ± 0.0524	0.6440 ± 0.1589

*Small (<4 ml), Medium (4–18 ml), Big (>18 ml).

Once the full brain is segmented into 135 labels, those labels are grouped together to provide information at different anatomical scales. Specifically, eight different tissue labels are generated. Dice results are summarized in Table 4.

**Table 4:** Proposed method dice results for each brain tissue.

**CSF**	**cGM**	**cWM**	**sGM**
0.9006 ± 0.0307	0.9543 ± 0.0144	0.9669 ± 0.0131	0.9518 ± 0.0114
**ceGM**	**ceWM**	**BS**	**Lesion**
0.9644 ± 0.0172	0.9448 ± 0.0363	0.9693 ± 0.0137	0.6440 ± 0.1589

### Method Reproducibility

A very important feature for a measurement method is its reproducibility. To measure the reproducibility of the proposed method, we used a subset of our library. Specifically, we used 19 cases of the OASIS subset that were scanned and labeled twice. In this case, we have two sources of variability, which are related to the inter-image changes and manual labeling differences. To measure the reproducibility, we computed the dice coefficient between the two different segmentations (of each case and its repetition). This was done for both the manual segmentation (that we used as a reference) and the automatic one. Results are summarized in Table 5. As can be seen, the proposed method showed a slightly superior reproducibility than manual labeling.

**Table 5:** Proposed method dice results.

**Method**	**All labels**	**Cortical labels**	**Non-cortical labels**
vol2Brain	0.8405 ± 0.0181	0.8234 ± 0.0206	0.8856 ± 0.0158
Manual	0.8368 ± 0.0171	0.8198 ± 0.0200	0.8818 ± 0.0163

*The mean dice is evaluated on all the considered labels (135 without background)*.

### Method Comparison

It is difficult to compare the proposed method with similar state-of-the-art methods such as Freesurfer, as the labeling protocol is slightly different. For this reason, we have used as a freely available and well-known method called Joint Label Fusion as a reference (Wang and Yushkevich, 2013). This method is a state-of-the-art multi-atlas segmentation approach. To make it fully comparable, we used the corrected cases of our library as the atlas library. We summarized the results of the comparison in Table 6. We compared our proposed method with two versions of the JLF approach, one using an affine registered library (linear) and another using a non-linear registered library. It is worth to note the proposed method uses only a linearly registered library (i.e., no non-linear registration was used). As can be noticed, the proposed method was far superior to both versions.

**Table 6:** Proposed method dice results compared with the results of two versions of JLF method.

**Method**	**All labels**	**Cortical labels**	**Non-cortical labels**
vol2Brain	0.8262 ± 0.0257	0.7996 ± 0.0347	0.8969 ± 0.0157
JLF (linear)	0.7369 ± 0.0292	0.7016 ± 0.0337	0.8305 ± 0.0241
JLF (non-linear)	0.7591 ± 0.0252	0.7327 ± 0.0288	0.8291 ± 0.0228

### Computational Time

The proposed method takes around 20 min on average to complete the whole pipeline (including cortical thickness estimation and report generation). JLF method takes around only 2 h for structure segmentations without cortical thickness estimation (excluding the preprocessing, which includes several hours of non-linear registration depending on the number of atlases used). Freesurfer normally takes around 6 h to perform the complete analysis (which also includes surface extraction).

## Discussion

We have presented a new pipeline for full brain segmentation (vol2Brain) that is able to segment the brain into 135 different regions in a very efficient and accurate manner. The proposed method also integrates these 135 regions to provide measures at different anatomical scales, including brain tissues and lobes. It also provides cortical thickness measurements per cortical structure and lobe displayed into an automatic report summarizing the results (refer to Appendix).

To create vol2Brain pipeline, we had to create a template library that integrates all the anatomical information needed to perform the labeling process. This was a long and laborious work, as the original library obtained from Neuromorphometrics did not meet the required quality and we had to invest a significant amount of time to make it ready to use. To create this library, we homogenized the image resolution, orientation, and size of the images, removed and relabeled inconsistent labels, and corrected systematic labeling errors. Besides, we extended the labeling protocol by adding external CSF and WM lesions. As a result, we generated a highly consistent and high-quality library that not only allowed to develop the current proposed pipeline but will also be a valuable resource for future developments.

The proposed method is based on patch-based multi-atlas label fusion technology. Specifically, we have used an optimized version of non-local label fusion called OPAL that efficiently finds patch matches needed to label each voxel in the brain by reducing the required time to label the full brain from hours to minutes. To further improve the results, we have used a patch-based error corrector, which has been previously used in other segmentation problems such as hippocampus subfield labeling (Romero et al., 2017) or cerebellum lobules (Carass et al., 2018).

We measured the results of the proposed pipeline using a LOO methodology and achieved an average dice value of 0.8262. This result was obtained from four different sub-datasets ranking from 0.7831 to 0.8353 showing a good generalization of the proposed method. This result was quite close to the manual intraobserver accuracy that was estimated as 0.8363 using a reduced dataset. We also compared the proposed method with a related currently available state-of-the-art method for full brain labeling. We demonstrated that vol2Brain was not only far superior to the linear (0.8262 vs. 0.7369) and nonlinear (0.8262 vs. 0.7591) versions of JLF method but also more efficient with a temporal cost of minutes compared with hours.

The proposed vol2Brain pipeline is already available through our volBrain platform (https://volbrain.upv.es). As compared to the rest of the volBrain platform pipelines, this pipeline receives an anonymized and compressed nifti file (a T1-weighted image in the case of vol2Brain) through the website and reports the results 20 min later by sending an email to the user. The user can also download the segmentation nifti files through the user area of volBrain platform (an example of the pdf report is shown in Appendix).

We hope that the accuracy and efficiency of the proposed method and the ease of use through the volBrain platform will boost the anatomical analysis of the normal and pathological brain (especially on those cases with WM lesions).

## Conclusion

In this study, we present a novel pipeline to densely segment the brain and to provide measurements of different features at different anatomical scales in an accurate and efficient manner. The proposed pipeline has been compared with a state-of-the-art-related method showing competitive results in terms of accuracy and computational time. Finally, we hope that the online accessibility of the proposed pipeline will facilitate the access of any user around the world to the proposed pipeline making their MRI data analysis simpler and more efficient.

## Data Availability Statement

The datasets presented in this article are not publicly available because the dataset is currently protected by a license. Requests to access the datasets should be directed to the corresponding author.

## Author Contributions

JM and PC designed and implemented the software. JR helped in the experiments and coding. RV-H, GR, MI-V, and FA helped in the library definition and report generation. All authors writed and reviewed the paper. All authors contributed to the article and approved the submitted version.

## Funding

This research was supported by the Spanish DPI2017-87743-R grant from the Ministerio de Economia, Industria y Competitividad of Spain. This work was benefited from the support of the project DeepvolBrain of the French National Research Agency (ANR-18-CE45-0013). This study was achieved within the context of the Laboratory of Excellence TRAIL ANR-10-LABX-57 for the BigDataBrain project.

## Conflict of Interest

The authors declare that the research was conducted in the absence of any commercial or financial relationships that could be construed as a potential conflict of interest.

## Publisher's Note

All claims expressed in this article are solely those of the authors and do not necessarily represent those of their affiliated organizations, or those of the publisher, the editors and the reviewers. Any product that may be evaluated in this article, or claim that may be made by its manufacturer, is not guaranteed or endorsed by the publisher.
