# Supplementary material for: vol2Brain: A New Online Pipeline for Whole Brain MRI Analysis
Source: Front Neuroinform. 2022 May 24;16:862805. doi: 10.3389/fninf.2022.862805 (PMC9171328; doi:10.3389/fninf.2022.862805)

**Appendix**

In table 1 a list of the 135 labels measured in vol2Brain pipeline is shown.

*Table 1. List of the structures labelled in vol2Brain pipeline.*

| **Index** | **Label Number** | **Label Name** |
| --- | --- | --- |
| 1 | 1 | CSF |
| 2 | 4 | 3rd Ventricle |
| 3 | 11 | 4th Ventricle |
| 4 | 23 | Right Accumbens |
| 5 | 30 | Left Accumbens |
| 6 | 31 | Right Amygdala |
| 7 | 32 | Left Amygdala |
| 8 | 35 | Brain Stem |
| 9 | 36 | Right Caudate |
| 10 | 37 | Left Caudate |
| 11 | 38 | Right Cerebellum Exterior |
| 12 | 39 | Left Cerebellum Exterior |
| 13 | 40 | Right Cerebellum White Matter |
| 14 | 41 | Left Cerebellum White Matter |
| 15 | 44 | Right Cerebral White Matter |
| 16 | 45 | Left Cerebral White Matter |
| 17 | 47 | Right Hippocampus |
| 18 | 48 | Left Hippocampus |
| 19 | 49 | Inferior Right Lateral Ventricle |
| 20 | 50 | Inferior Left Lateral Ventricle |
| 21 | 51 | Right Lateral Ventricle |
| 22 | 52 | Left Lateral Ventricle |
| 23 | 53 | Right Lesion |
| 24 | 54 | Left Lesion |
| 25 | 55 | Right Pallidum |
| 26 | 56 | Left Pallidum |
| 27 | 57 | Right Putamen |
| 28 | 58 | Left Putamen |
| 29 | 59 | Right Thalamus |
| 30 | 60 | Left Thalamus |
| 31 | 61 | Right Ventral DC |
| 32 | 62 | Left Ventral DC |
| 33 | 71 | Cerebellar Vermal Lobules I-V |
| 34 | 72 | Cerebellar Vermal Lobules VI-VII |
| 35 | 73 | Cerebellar Vermal Lobules VIII-X |
| 36 | 75 | Left Basal Forebrain |
| 37 | 76 | Right Basal Forebrain |
| 38 | 100 | Right anterior cingulate gyrus |
| 39 | 101 | Left anterior cingulate gyrus |
| 40 | 102 | Right anterior insula |
| 41 | 103 | Left anterior insula |
| 42 | 104 | Right anterior orbital gyrus |
| 43 | 105 | Left anterior orbital gyrus |
| 44 | 106 | Right angular gyrus |
| 45 | 107 | Left angular gyrus |
| 46 | 108 | Right calcarine cortex |
| 47 | 109 | Left calcarine cortex |
| 48 | 112 | Right central operculum |
| 49 | 113 | Left central operculum |
| 50 | 114 | Right cuneus |
| 51 | 115 | Left cuneus |
| 52 | 116 | Right entorhinal area |
| 53 | 117 | Left entorhinal area |
| 54 | 118 | Right frontal operculum |
| 55 | 119 | Left frontal operculum |
| 56 | 120 | Right frontal pole |
| 57 | 121 | Left frontal pole |
| 58 | 122 | Right fusiform gyrus |
| 59 | 123 | Left fusiform gyrus |
| 60 | 124 | Right gyrus rectus |
| 61 | 125 | Left gyrus rectus |
| 62 | 128 | Right inferior occipital gyrus |
| 63 | 129 | Left inferior occipital gyrus |
| 64 | 132 | Right inferior temporal gyrus |
| 65 | 133 | Left inferior temporal gyrus |
| 66 | 134 | Right lingual gyrus |
| 67 | 135 | Left lingual gyrus |
| 68 | 136 | Right lateral orbital gyrus |
| 69 | 137 | Left lateral orbital gyrus |
| 70 | 138 | Right middle cingulate gyrus |
| 71 | 139 | Left middle cingulate gyrus |
| 72 | 140 | Right medial frontal cortex |
| 73 | 141 | Left medial frontal cortex |
| 74 | 142 | Right middle frontal gyrus |
| 75 | 143 | Left middle frontal gyrus |
| 76 | 144 | Right middle occipital gyrus |
| 77 | 145 | Left middle occipital gyrus |
| 78 | 146 | Right medial orbital gyrus |
| 79 | 147 | Left medial orbital gyrus |
| 80 | 148 | Right postcentral gyrus medial segment |
| 81 | 149 | Left postcentral gyrus medial segment |
| 82 | 150 | Right precentral gyrus medial segment |
| 83 | 151 | Left precentral gyrus medial segment |
| 84 | 152 | Right superior frontal gyrus medial segment |
| 85 | 153 | Left superior frontal gyrus medial segment |
| 86 | 154 | Right middle temporal gyrus |
| 87 | 155 | Left middle temporal gyrus |
| 88 | 156 | Right occipital pole |
| 89 | 157 | Left occipital pole |
| 90 | 160 | Right occipital fusiform gyrus |
| 91 | 161 | Left occipital fusiform gyrus |
| 92 | 162 | Right opercular part of the inferior frontal gyrus |
| 93 | 163 | Left opercular part of the inferior frontal gyrus |
| 94 | 164 | Right orbital part of the inferior frontal gyrus |
| 95 | 165 | Left orbital part of the inferior frontal gyrus |
| 96 | 166 | Right posterior cingulate gyrus |
| 97 | 167 | Left posterior cingulate gyrus |
| 98 | 168 | Right precuneus |
| 99 | 169 | Left precuneus |
| 100 | 170 | Right parahippocampal gyrus |
| 101 | 171 | Left parahippocampal gyrus |
| 102 | 172 | Right posterior insula |
| 103 | 173 | Left posterior insula |
| 104 | 174 | Right parietal operculum |
| 105 | 175 | Left parietal operculum |
| 106 | 176 | Right postcentral gyrus |
| 107 | 177 | Left postcentral gyrus |
| 108 | 178 | Right posterior orbital gyrus |
| 109 | 179 | Left posterior orbital gyrus |
| 110 | 180 | Right planum polare |
| 111 | 181 | Left planum polare |
| 112 | 182 | Right precentral gyrus |
| 113 | 183 | Left precentral gyrus |
| 114 | 184 | Right planum temporale |
| 115 | 185 | Left planum temporale |
| 116 | 186 | Right subcallosal area |
| 117 | 187 | Left subcallosal area |
| 118 | 190 | Right superior frontal gyrus |
| 119 | 191 | Left superior frontal gyrus |
| 120 | 192 | Right supplementary motor cortex |
| 121 | 193 | Left supplementary motor cortex |
| 122 | 194 | Right supramarginal gyrus |
| 123 | 195 | Left supramarginal gyrus |
| 124 | 196 | Right superior occipital gyrus |
| 125 | 197 | Left superior occipital gyrus |
| 126 | 198 | Right superior parietal lobule |
| 127 | 199 | Left superior parietal lobule |
| 128 | 200 | Right superior temporal gyrus |
| 129 | 201 | Left superior temporal gyrus |
| 130 | 202 | Right temporal pole |
| 131 | 203 | Left temporal pole |
| 132 | 204 | Right triangular part of the inferior frontal gyrus |
| 133 | 205 | Left triangular part of the inferior frontal gyrus |
| 134 | 206 | Right transverse temporal gyrus |
| 135 | 207 | Left transverse temporal gyrus |

**Report example**

Example report of the vol2Brain pipeline. The report summarizes volumetric/asymmetry and thickness information at different scales as long as different captures of the results as a visual quality control.


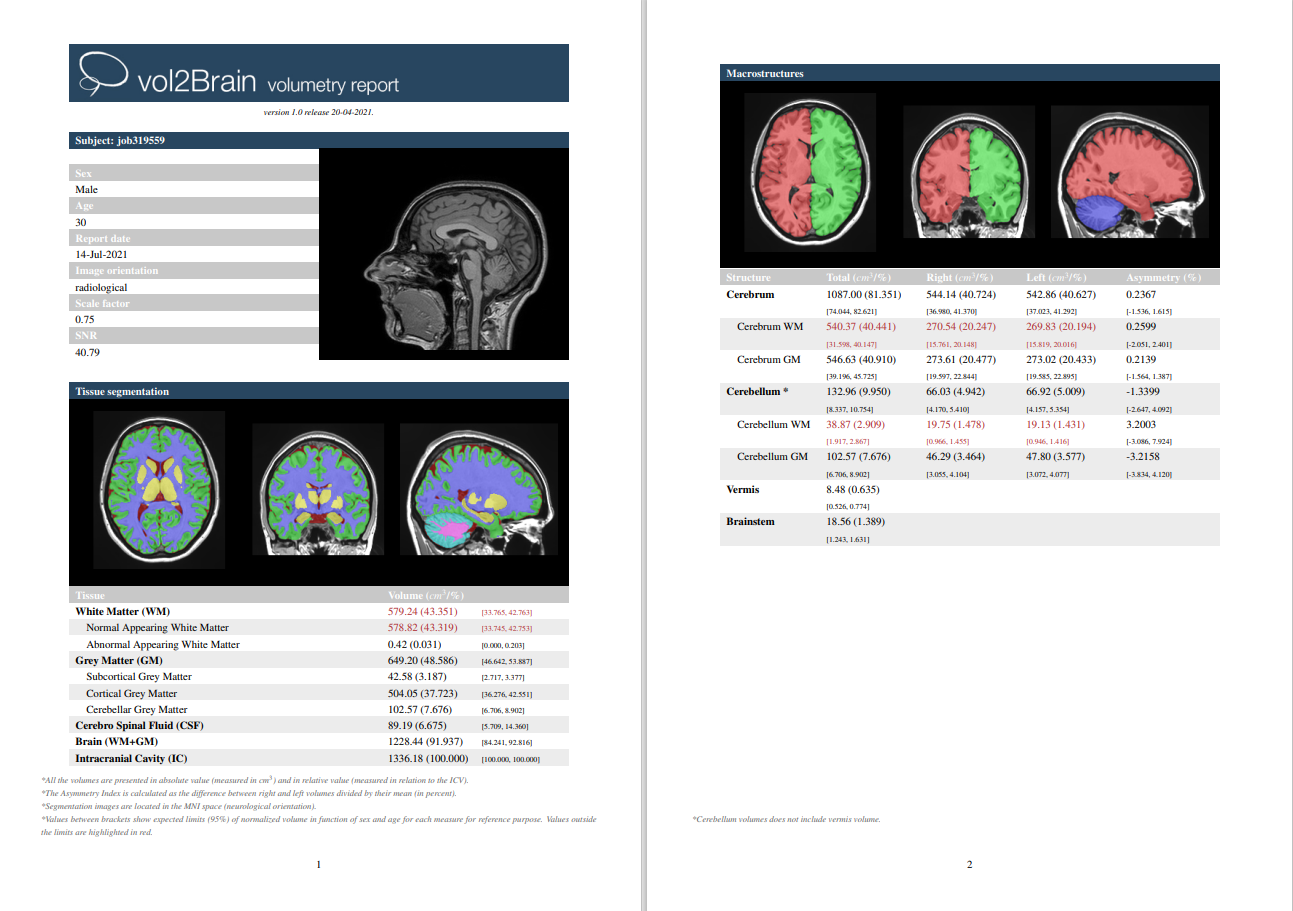


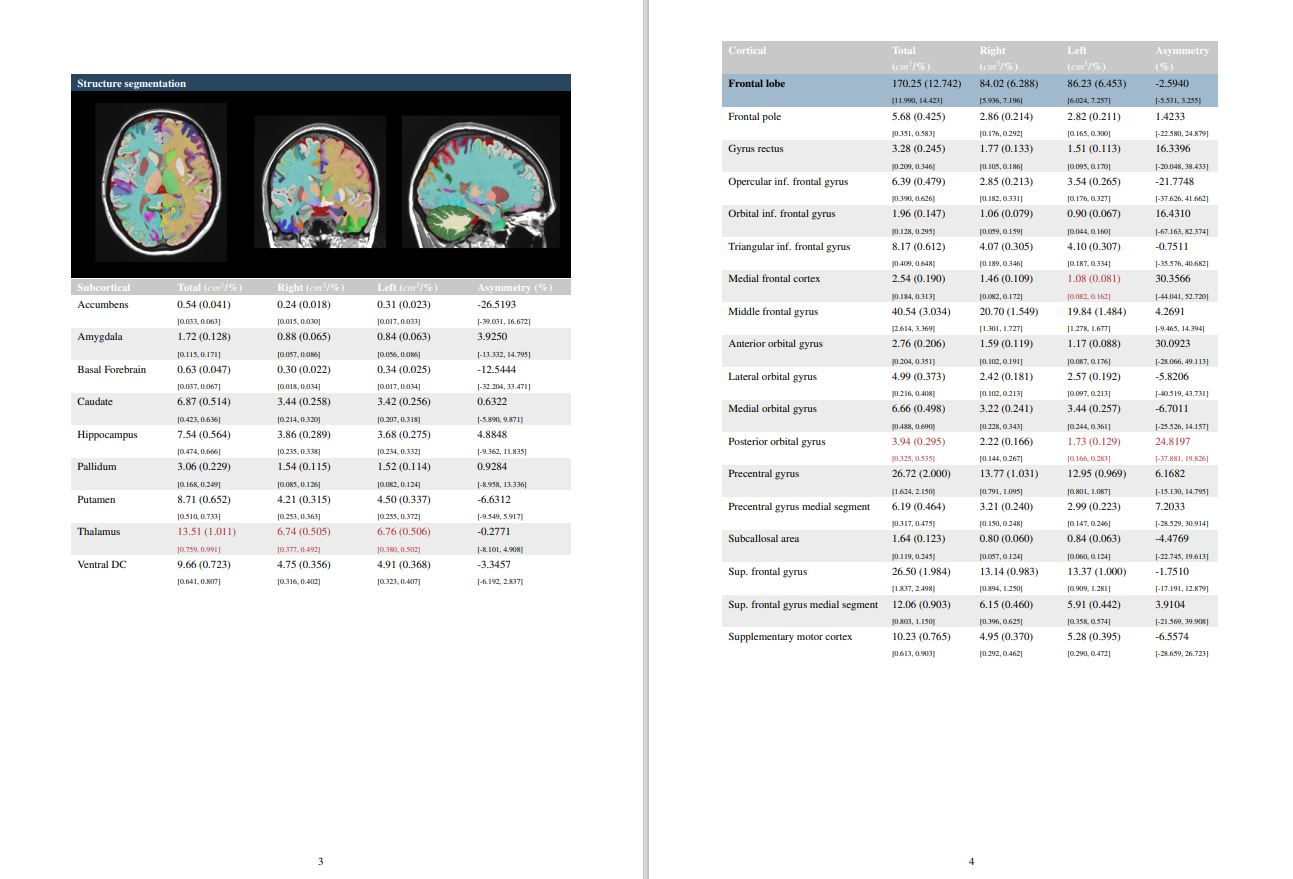


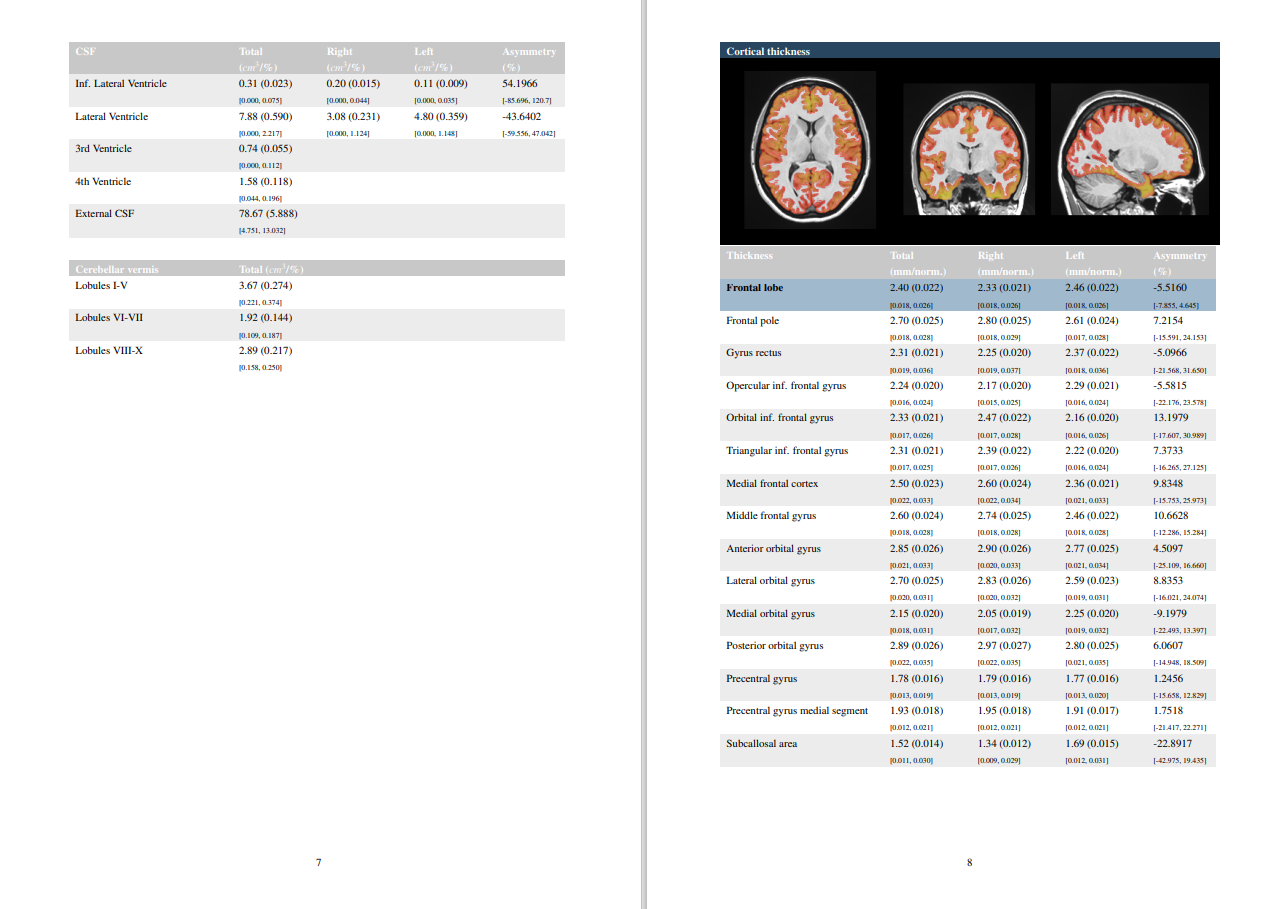

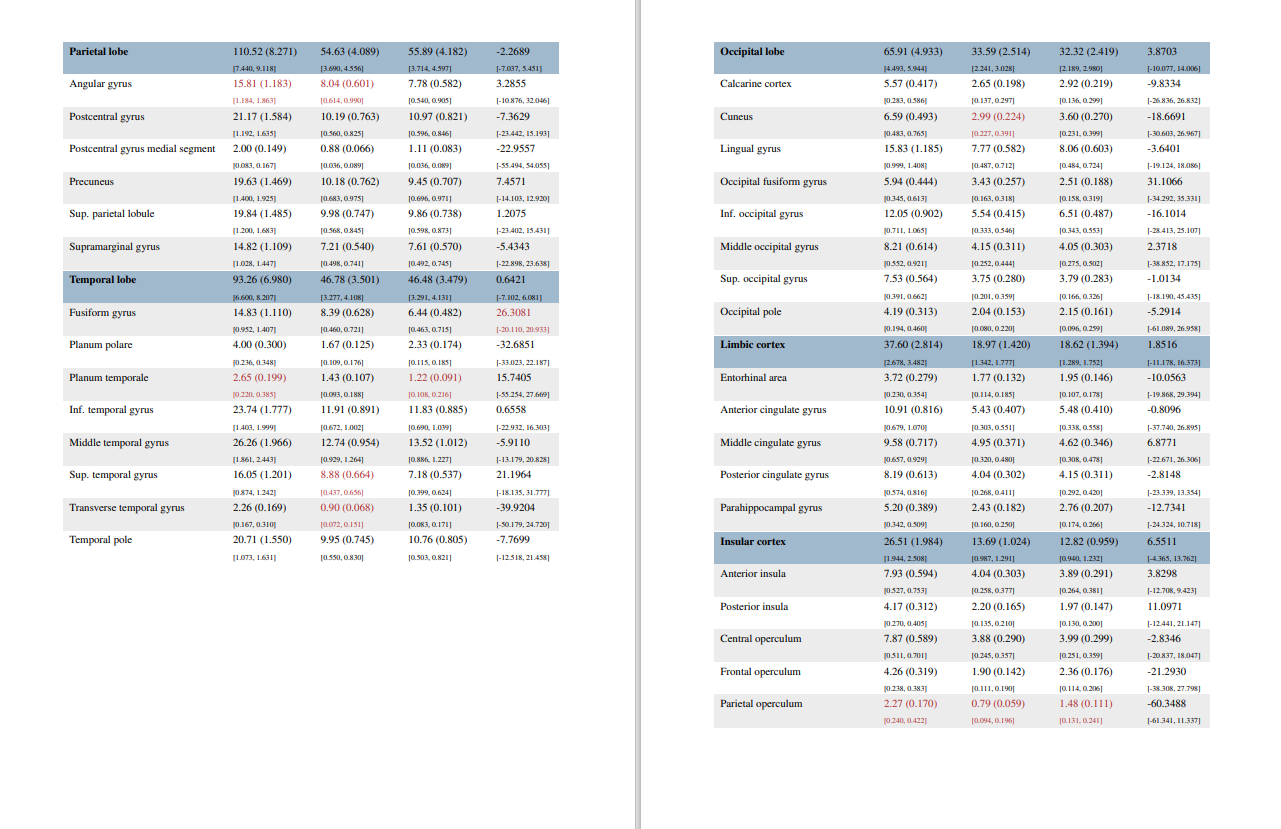


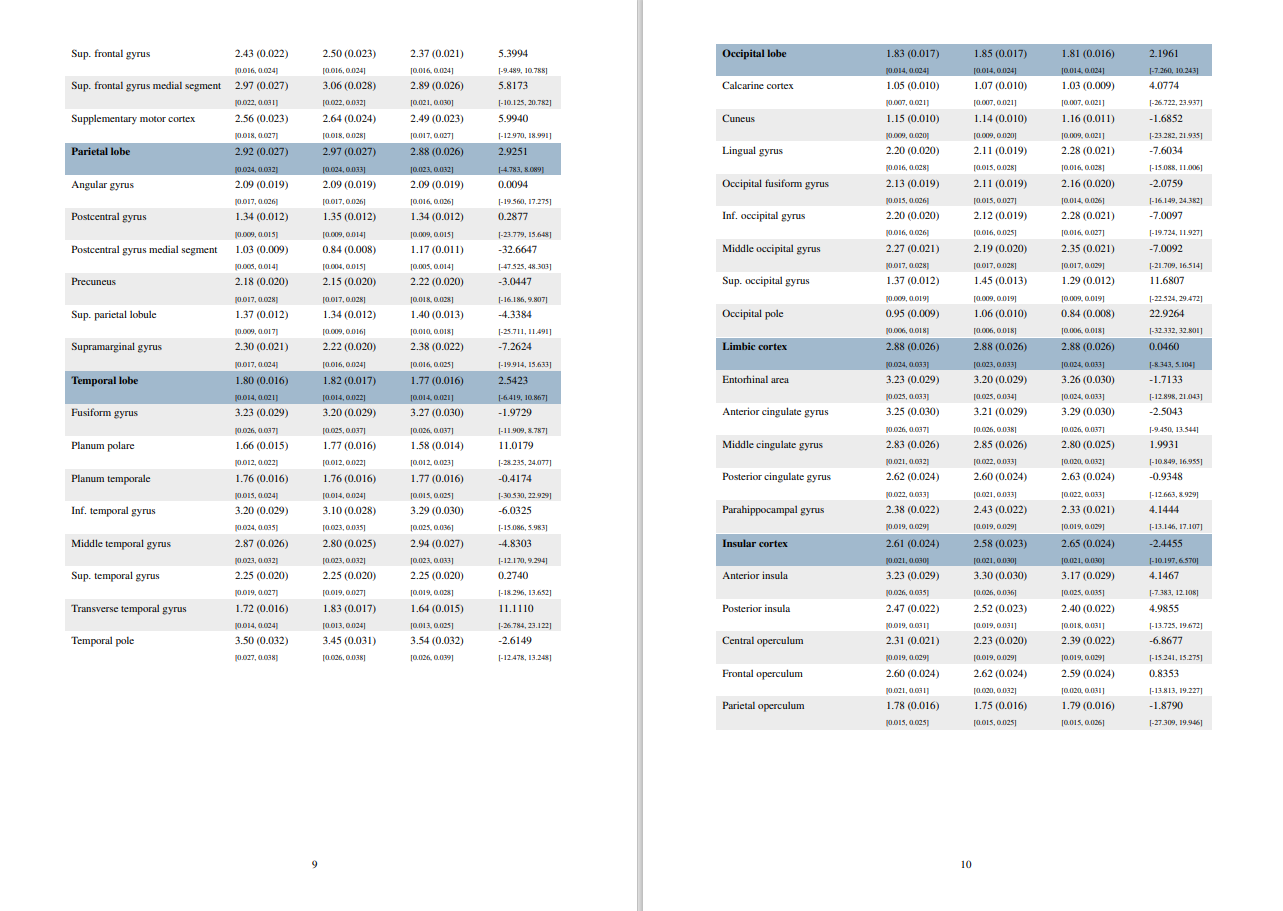

Supplement: Supplementary file 1 [file Data_Sheet_1.docx]
